# Symptoms and Medication Use in Children with Asthma and Traffic-Related Sources of Fine Particle Pollution

**DOI:** 10.1289/ehp.0800335

**Published:** 2009-03-31

**Authors:** Janneane F. Gent, Petros Koutrakis, Kathleen Belanger, Elizabeth Triche, Theodore R. Holford, Michael B. Bracken, Brian P. Leaderer

**Affiliations:** 1 Center for Perinatal, Pediatric and Environmental Epidemiology, Department of Epidemiology and Public Health, Yale University School of Medicine, New Haven, Connecticut, USA; 2 Harvard University School of Public Health, Boston, Massachusetts, USA

**Keywords:** childhood asthma, fine particle pollution, PM_2.5_, respiratory morbidity, source apportionment, traffic pollution

## Abstract

**Background:**

Exposure to ambient fine particles [particulate matter ≤ 2.5 μm diameter (PM_2.5_)] is a potential factor in the exacerbation of asthma. National air quality particle standards consider total mass, not composition or sources, and may not protect against health impacts related to specific components.

**Objective:**

We examined associations between daily exposure to fine particle components and sources, and symptoms and medication use in children with asthma.

**Methods:**

Children with asthma (*n =* 149) 4–12 years of age were enrolled in a year-long study. We analyzed particle samples for trace elements (X-ray fluorescence) and elemental carbon (light reflectance). Using factor analysis/source apportionment, we identified particle sources (e.g., motor vehicle emissions) and quantified daily contributions. Symptoms and medication use were recorded on study diaries. Repeated measures logistic regression models examined associations between health outcomes and particle exposures as elemental concentrations and source contributions.

**Results:**

More than half of mean PM_2.5_ was attributed to traffic-related sources motor vehicles (42%) and road dust (12%). Increased likelihood of symptoms and inhaler use was largest for 3-day averaged exposures to traffic-related sources or their elemental constituents and ranged from a 10% increased likelihood of wheeze for each 5-μg/m^3^ increase in particles from motor vehicles to a 28% increased likelihood of shortness of breath for increases in road dust. Neither the other sources identified nor PM_2.5_ alone was associated with increased health outcome risks.

**Conclusions:**

Linking respiratory health effects to specific particle pollution composition or sources is critical to efforts to protect public health. We associated increased risk of symptoms and inhaler use in children with asthma with exposure to traffic-related fine particles.

Fine particles [particles ≤ 2.5 μm diameter (PM_2.5_)] originate from both local and regional pollution sources. A large fraction of PM_2.5_ mass is associated with the combustion of fuels such as gasoline, diesel, coal, oil, and wood. PM_2.5_ mass also includes crustal material (road dust and soil particles), sea salt, and biological particles. Factor analysis and source apportionment techniques [Brown et al. 2007; U.S. Environmental Protection Agency (EPA) 2008; Watson et al. 2002] have been used for many years to help policy regulators identify sources of particle pollution. These techniques have been used to investigate associations between sources of particle pollution and health effects including studies of adult mortality (Laden et al. 2000; Mar et al. 2000; Ozkaynak and Thurston 1987; Taino et al. 2005), adult emergency department visits (Sarnat et al. 2008), and adult and pediatric hospital admissions (Andersen et al. 2007). Recently, one study associated specific sources of fine particles with respiratory morbidity in adults with asthma (Penttinen et al. 2006).

PM_2.5_ mass concentration has been associated with adverse health effects in children, particularly children with asthma: increased respiratory morbidity and/or medication use (Gent et al. 2003; Johnston et al. 2006; Mar et al. 2004; Rabinovitch et al. 2006; Ranzi et al. 2004; Rodriguez et al. 2007; Slaughter et al. 2003); increased number of doctor visits for respiratory illness (Chardon et al. 2007; Hertz-Picciotto et al. 2007; Pino et al. 2004); and decreased lung function (Delfino et al. 2004; Hong et al. 2007; Lewis et al. 2005; Moshammer et al. 2006; Trenga et al. 2006). Linking respiratory health effects to specific particle pollution sources is critical to efforts to develop cost-effective strategies to protect public health, particularly the respiratory health of vulnerable members of the population such as children with asthma.

Annual PM_2.5_ levels at a monitoring site in New Haven, Connecticut, where subjects in the present study live, exceed the U.S. EPA annual standard of 15 μg/m^3^ (U.S. EPA 2009). We examined associations between daily exposure to PM_2.5_ components and sources identified through factor analysis and source apportionment, and daily symptoms and medication use in children with asthma. Some of the results of this study have been previously reported in the form of an abstract (Gent et al. 2008).

## Methods

### Subjects

Subjects were drawn from a larger study population 4–12 years of age when enrolled between 2000 and 2003. Eligibility criteria were physician-diagnosed asthma and symptoms or medication use in the previous 12 months. Subjects in the present analysis (*n =* 149) were restricted to those residing in New Haven County within 30 km of the air quality monitor. Study participants included siblings with asthma (one per family; *n =*88, 59%) of infants enrolled in a birth cohort to study the development of asthma (Belanger et al. 2003), patients recruited from the Yale Pediatric Asthma Clinic (*n =*43, 29%), and children with asthma identified through a survey on childhood asthma conducted in the New Haven public schools (*n =* 18, 12%). In addition to abundant mobile sources of air pollution (e.g., interstate highways 95, 91, 84), New Haven is home to stationary sources including small manufacturing enterprises, power plants, and a harbor handling ships that deliver heating oil for New England (U.S. EPA 2008). New Haven County, population 824,008, covers an area of 2,233 km^2^ (863 mi^2^) (U.S. Census Bureau 2000). Yale University Human Investigation Committee approved the study, and all respondents (mothers of study subjects) gave informed consent prior to participation.

### Data collection

During the enrollment visit, a research assistant administered a questionnaire to the child’s mother to collect demographic, medical history, and home environment information. Mothers recorded daily symptoms [wheeze, persistent cough (defined as coughing throughout the day), shortness of breath, chest tightness, night symptoms] and medication use (rescue medication including bronchodilators, and maintenance medication including steroids, cromolyn sodium, and leukotriene inhibitors) on study diaries and reported this information during monthly telephone interviews. Dates away from New Haven during the follow-up period were reported at an exit interview and were not used in the analysis.

### Exposure measurements

#### Filter analysis

PM_2.5_ filter samples (*n* = 1,181) were collected between 1 August 2000 and 3 February 2004 from the Stiles Street monitoring site (98% of the filters) or the State Street site (within 3 km). Filters were analyzed by X-ray fluorescence (XRF) (Desert Research Institute, Reno, Nevada) (Watson et al. 1999). A list of the 51 elements analyzed is presented in the Supplemental Material, Table 1 (available online at http://www.ehponline.org/members/2009/0800335/suppl.pdf).

#### Light reflectance analysis

Before XRF, elemental carbon (EC) concentrations were determined using an optical reflectance technique (Cyrys et al. 2003; Janssen et al. 2001; Kinney et al. 2000). See Supplemental Material for further details (available online at http://www.ehponline.org/members/2009/0800335/suppl.pdf).

#### Source apportionment

We analyzed daily concentrations of 17 elements [see Supplemental Material, Table 1 (available online at http://www.ehponline.org/members/2009/0800335/suppl.pdf)] as well as EC using principal component analysis with an orthogonal (varimax) rotation in SAS (version 9.1; SAS Institute Inc., Cary, NC) (Kavouras et al. 2001; Thurston and Spengler 1985). We calculated daily scores for each factor by multiplying the normalized elemental concentrations by the respective standardized elemental scoring coefficient and factor, then summing across all component products. Scores were rescaled to concentrations in micrograms per cubic meter by regressing total PM_2.5_ concentration on factor scores and obtaining the product of each factor score with its regression coefficient. Rescaled factor scores, or daily source contributions, were used as exposures in the health effects analyses.

Six sources of PM_2.5_ were identified based on loadings (or correlations) of elements on each of the six factors determined by factor analysis. The factor with high loadings for EC and trace elements zinc, lead, and copper was attributed to motor vehicle emissions. The factor road dust was associated with elements of terrestrial origin such as silicon, iron, aluminum, and calcium as well as barium, which is a tracer for tire wear particles. The sulfur factor represents particles from regional sources and was highly correlated with the elements sulfur and phosphorus. The factor with a high potassium loading was attributed to biomass burning. The oil factor represents oil combustion emissions from power plants and home heating and was characterized by the presence of vanadium and nickel. Finally, the sea salt factor was associated with the elements sodium and chlorine.

### Data analysis

The association between daily elemental concentrations or source contributions and daily respiratory symptoms and rescue medication use was examined with repeated measures logistic regression (SAS Institute Inc.). Models used generalized estimating equations (GEE) specifying a 1-day lag autoregressive structure (AR1) for the health outcomes correlation matrices. All models were adjusted for season using sine and cosine functions of date to capture annual cycles [i.e., cos((date/365) × 2π) and sin((date/365) × 2π)], day of week (for weekly cycles), and date (to control for time trends over the 4-year course of the study). Because sources were uncorrelated by design (orthogonal rotation of factors), all six sources were included simultaneously in health effects models. Gaseous co-pollutants (nitrogen dioxide, carbon monoxide, sulfur dioxide, ozone) were examined separately in models that also included all six sources. Models included year-round data except for the co-pollutant model with O_3_, which is monitored only in warm months. Health effects of elements included in the factor analysis, all six sources, and PM_2.5_ were examined for same-day exposures [lag0 (L0)] as well as exposures lagged by 1 or 2 days (L1, L2) or the mean of lagged days 0–2 (L02). Tests for goodness of fit were performed using the Hosmer–Lemeshow statistic for logistic regression.

## Results

**Figure 1:**
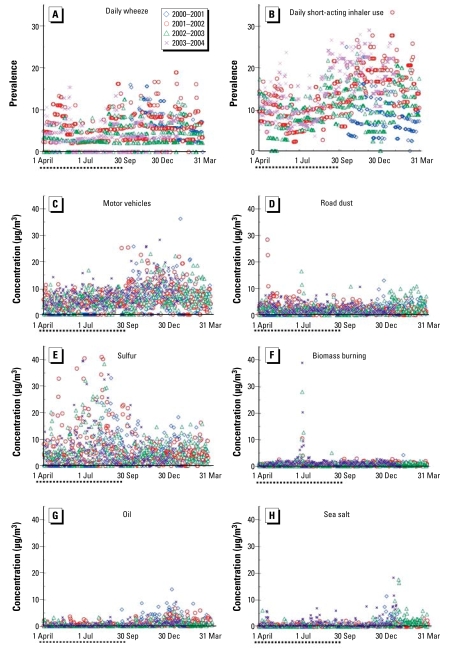
Daily prevalence of wheeze (*A*) and short-acting inhaler use (*B*). Daily PM_2.5_ source concentrations of motor vehicle (*C*), road dust (*D*), sulfur (*E*), biomass burning (*F*), oil (*G*), and sea salt (*H*). Dotted line at left of *x*-axis indicates warmer months (April–September). *n* = 149 children, New Haven, Connecticut, August 2000–January 2004.

Demographic characteristics of study participants are shown in Table 1. Most subjects were male (60%), ≥ 8 years of age (53%) (mean ± SD = 8 ± 2 years), and self-reported as white (56%). More than one-third of the adult respondents had a college degree (36%). Environmental tobacco smoke exposure was reported in 6% of the homes. Subjects participated for a mean of 313 ± 92 days [median 366; interquartile range (IQR) 54 days]. The distribution of daily symptoms and medication use is shown in Table 2. The most frequently experienced symptom was persistent cough (reported by 86%), followed by wheeze (76%), which was experienced by subjects for a median of 19 days (IQR 29) or 8 days (IQR 24), respectively, of the year of follow-up. Subjects experienced shortness of breath or chest tightness (reported by two-thirds of the subjects) less frequently for a median of 4 (IQR 14) days each, and used short-acting inhalers (reported by 80%) for a median of 18 (IQR 47) days. Nearly one-third of the subjects had moderate to severe persistent asthma during the year-long follow-up study (Table 2). All subjects resided 0.9–27 km from the air pollution monitor site (mean ± SD = 10.2 ± 8.4 km). The mean number of subjects under observation each day was 36 ± 8 and ranged from 10 to 49. The mean number of subjects experiencing symptoms or using short-acting inhalers each day was as follows: shortness of breath or chest tightness 3 ± 3%; wheeze 5 ± 4%; persistent cough 8 ± 5%; and inhaler use 12 ± 6% (Figure 1A,B).

Mean level of PM_2.5_ during the study was 17.0 ± 9.8 μg/m^3^, which is above the U.S. annual standard for fine particles of 15 μg/m^3^ (U.S. EPA 2009). The contributions from each source to PM_2.5_ mass and to elemental components of PM_2.5_ are given in Table 3. The mean estimated and measured mass are given as well as the percent root mean square error (%RMSE), defined as 100 × (|estimated concentration – measured concentration|/measured concentration). Elemental components of PM_2.5_ are grouped according to the source with which they were most strongly associated. For example, the motor vehicle source contributed 62% of the estimated mean EC mass (961.3 of 1554.8 ng/m^3^) and 77% of the mean Zn mass (22.3 of 29.0 ng/m^3^). On average, over the 4 years of the study, over half of daily PM_2.5_ came from the two traffic-related sources motor vehicles [median (IQR) 42.5% (26.7%)] and road dust [12.3% (13.8%)] (bottom of Table 2). An additional 28.7% (30.3%) of daily PM_2.5_ came from regional sulfur sources.

The associations between PM_2.5_ elemental components and daily symptoms or medication use are presented in Table 4. Individual elements in fine particle mass may come from more than one source but are listed in Table 4 according to the source with which they were most strongly associated (Table 3). Associations for same-day exposure (L0) and exposures averaged over the same day and previous 2 days (L02) are shown in Table 4. Exposures lagged by 1 or 2 days (L1, L2) are included in Table 2 in the Supplemental Material (available online at http://www.ehponline.org/members/2009/0800335/suppl.pdf). In general, trace elements originating from the motor vehicle, road dust, biomass burning, and oil sources are associated with symptoms and/or medication use. For example, a significantly increased likelihood of wheeze, shortness of breath, chest tightness, or short-acting inhaler use is associated with each 1,000-ng/m^3^ increase in EC. The strongest associations were found for the 3-day averaged exposures to the elemental constituents of road dust. No associations were found between individual elements associated with sulfur or sea salt sources and symptoms or medication use. Total PM_2.5_ was not significantly associated with any symptoms or medication use [see Supplemental Material, Table 3 (available online at http://www.ehponline.org/members/2009/0800335/suppl.pdf)].

Daily source contributions to PM_2.5_ are illustrated for the 4 years of the study in Figure 1C–1H. Results of logistic regression models examining associations between health outcomes and PM_2.5_ sources are shown in Table 5 for same-day exposures (Table 5, L0 model) and exposures averaged over the same day and previous 2 days (Table 5, L02 model). Source exposures lagged by 1 or 2 days (L1, L2) are included in the Supplemental Material, Table 3 (available online at http://www.ehponline.org/members/2009/0800335/suppl.pdf). For same-day or 3-day averaged exposures, each 5-μg/m^3^ increase in PM_2.5_ mass concentration coming from either motor vehicles or road dust is consistently associated with increases in the likelihood of respiratory symptoms or inhaler use (Table 5). Reduced likelihood of wheeze or inhaler use is associated with 5-μg/m^3^ increases in the same day, but not for lagged exposures to the sulfur source. Significant reductions in the likelihood of wheeze were also associated with exposures to the biomass burning source.

The effect of adding a gaseous co-pollutant (NO_2_, CO, SO_2_, or O_3_) to the source exposure model is shown for wheeze and short-acting inhaler use in Table 6. For example, the addition of any of the gaseous co-pollutants to the source exposure model reduces the effect of the motor vehicle source on wheeze. Interestingly, in the source model that also includes NO_2_ (another marker for traffic), each 20-ppb increase in NO_2_ increases the likelihood of wheeze by 8% (*p* = 0.09). No significant associations were seen in co-pollutant models between exposure and likelihood of persistent cough or chest tightness.

## Discussion

Results of the source apportionment analysis suggest that over half of the mean PM_2.5_ can be attributed to traffic sources motor vehicles and road dust, and that daily exposure to these sources is associated with increased risk of respiratory symptoms and inhaler use in children with asthma. Risks remain in models that include all six PM_2.5_ sources as well as one gaseous co-pollutant. Of co-pollutants added to the model one at a time, NO_2_, which is a marker for traffic (Grieshop et al. 2006), was also found to be an independent risk factor for increased wheeze (Table 6).

In single-element analyses, EC, also considered a marker for traffic (Grieshop et al. 2006; Sarnat et al. 2008), is itself associated with increased symptoms (wheeze, shortness of breath, chest tightness) and medication use (Table 4). Of the EC measured in PM_2.5_, 88% was contributed by the traffic-related sources: 62% from motor vehicles and 26% from road dust (Table 3). Among elements associated with the road dust source, Ca appears to have the strongest association with symptoms (Table 4). Numerous studies have found that the composition of Ca in road dust is higher than that in the earth’s crust (Grieshop et al. 2006). Results from tunnel and chassis dynamometer studies have shown that Ca is emitted from light and heavy-duty vehicles and is possibly associated with the combustion of motor oil (Ning et al. 2008). The stronger associations with fine-particle Ca compared with other major terrestrial elements such as Al and Si suggests that it may be a better tracer of urban road dust.

A negative association was observed between biomass burning and wheeze (Table 5). Potassium, the tracer element for biomass burning source and an element used in firework propellant mixture, was present at high concentrations when Fourth of July displays took place (Moreno et al. 2007). When the analysis was rerun without the days with the five highest potassium values [all > 1,000 ng/m^3^ and all occurring in July (4 July 20014 July 20024 July 2003; 5 July 2003; 7 July 2002) (Figure 1F)], the negative association seen previously for wheeze remains but is no longer significant [odds ratio (OR) 0.85; *p* = 0.29]. Of note is the high level of potassium measured on 7 July 2002, which is attributable not to fireworks but to a smoke plume from a massive Canadian forest fire (Sigler et al. 2003). For our subjects, symptoms and medication use are at their lowest in summer and highest in winter (Figure 1A,B), and if these children with asthma were kept away from fireworks displays and/or kept indoors during the smoke plume days, this may have resulted in the apparent protective association with potassium levels.

Subjects who were in follow-up during the summer of 2001 also contributed data to an earlier analysis of O_3_ and respiratory health in southern New England (Gent et al. 2003). In that analysis, we found O_3_ exposure to be a risk for increased respiratory symptoms, but only among children taking asthma maintenance medication (e.g., corticosteroids). The model with PM_2.5_ sources and O_3_ shown in Table 6 shows O_3_ to be a risk factor increasing the likelihood of wheeze by 8% (*p* = 0.08), but this model includes data from all children living in New Haven regardless of maintenance medication status. Reanalysis stratified by maintenance medication use showed that every 50-ppb increase in O_3_ (8-hr average) resulted in a larger risk of wheeze for users of maintenance medication (*n =*76) [OR = 1.29; 95% confidence interval (CI), 0.99–1.68; *p* = 0.06] than for nonusers (*n =*63) (OR = 1.10; 95% CI, 0.77–1.57; *p* = 0.60). Comparable results from the earlier report are given for a 50-ppb increase in 1-hr O_3_: For children on maintenance medication (*n =* 130), the likelihood of wheeze increases by 35% (OR = 1.35; 95% CI, 1.11–1.65) (Gent et al. 2003).

Although exposure to total PM_2.5_ mass has itself been shown to be a significant risk factor for adverse respiratory outcomes among children (Johnston et al. 2006; Mar et al. 2004; Rabinovitch et al. 2006; Ranzi et al. 2004; Rodriguez et al. 2007; Slaughter et al. 2003), this is not always the case (Chimonas and Gessner 2007; Holguin et al. 2007). In a study of Medicaid claims for doctor visits for asthma in Anchorage, Alaska, the authors noted that the major constituent of their particles is geologic in origin (i.e., rock dust), and that annual levels of PM mass are low, 6 μg/m^3^ (95% CI, 0.5–7.0). The authors found no association between exposure to PM_2.5_ mass and health outcomes. On the other hand, significant effects were seen between increases in PM_10_ mass concentrations (PM with aerodynamic diameter < 10 μm) and increased visits for asthma and prescriptions for inhalers (Chimonas and Gessner 2007). In a study of asthmatic children and respiratory health in a busy, traffic-filled Mexican border town, Holguin et al. (2007) found no significant association between level of PM_2.5_ mass and respiratory health. However, using traffic as the exposure metric (calculated as traffic density in 50-m residential buffers) revealed a significant risk for increased respiratory symptoms with increasing traffic (OR = 1.58; 95% CI, 1.05–2.38) (Holguin et al. 2007).

Studies using proximity to traffic as an exposure metric suggest that children living near busy roads have impaired respiratory health. Large studies of schoolchildren in Munich (Nicolai et al. 2003) and Nottingham, United Kingdom (Venn et al. 2001), have found that proximity of residence (< 50–90 m) to heavily traveled roads is associated with increased risk of asthma symptoms. Studies of schoolchildren conducted in the Netherlands found increased health risks associated with proximity specifically to truck traffic and wheeze (Janssen et al. 2003), chronic respiratory symptoms (Van Vliet et al. 1997), and lung function (Brunekreef et al. 1997). Similar increased risk associations were found between black smoke (a proxy for diesel exhaust), measured at subjects’ schools, and respiratory outcomes. No significant associations were found between respiratory symptoms and volume of automobile traffic. The strongest associations were found for children living within 300 m of a highway (Brunekreef et al. 1997). A smaller study in Cincinnati, Ohio (Ryan et al. 2005), found an increased risk of wheezing in infants associated with stop-and-go bus and truck traffic within 100 m of the residence. All of these studies suggest that proximity to traffic, in particular high-volume truck traffic, has a negative impact on the respiratory health of children. Rather than a metric based on proximity to roads or traffic density, we used factor analysis and source apportionment techniques to examine exposures to potentially toxic portions of particle mass and the health of children residing in an area of significant mobile source activity.

One strength of this study is its longitudinal design. More than 46,000 subject-days of data were available for analyses (resulting in small CIs) where each subject served as his or her own control for potential confounders. Effect estimates from analyses where sex, age, ethnicity, parental education level, and exposure to environmental tobacco smoke were also included varied little from those shown in Table 6. For example, each 5-μg/m^3^ increase in exposure to motor vehicle source was associated with identical increased risks of wheeze and shortness of breath and inhaler use. Likewise, the risk estimates associated with exposure to road dust source were identical for persistent cough and inhaler use and slightly decreased for wheeze (OR = 1.09; 95% CI, 1.01–1.19) and shortness of breath (OR = 1.11; 95% CI, 1.02–1.21) (for comparison, see Table 5). Goodness-of-fit tests suggest that the models for wheeze and chest tightness are reasonably good fits to the data (Table 5). There were no systematic patterns to the lack of fit for models for persistent cough, shortness of breath, or short-acting inhaler use. However, because of the repeated measurements, observations were not independent in any of the models, which may affect the interpretation of the Hosmer–Lemeshow statistic. It is possible that the more frequently reported events of persistent cough and short-acting inhaler use (Table 2) may be associated with ambient air pollution in combination with other factors (e.g., activity level) not included in the current study.

One limitation of our study is the lack of co-located EC calibration measurements. Previous studies have used thermal optical reflectance (TOR) to measure EC collected on quartz filters in co-located monitors. TOR, a relatively costly but accurate measure of EC, is used to calibrate EC estimated by the much less expensive light reflectance measures on Teflon filters. We assumed a factor of 1 based on calibration factors reported in other studies examining air pollution in urban settings (Cyrys et al. 2003; Kinney et al. 2000) and on our own results of TOR EC measurements on 30, co-located quartz filters [see Supplemental Material (available online at http://www.ehponline.org/members/2009/0800335/suppl.pdf)].

Another limitation to our study is our reliance on a central site monitor. It is likely that measurements from this site, located on an on-ramp to I-95 at the southern edge of New Haven County, represent the highest end of the exposure distribution. Exposure error for regional sources, for example, sulfur, is probably low because of its homogeneity within the study area. On the other hand, local traffic exposures vary across the study area, which may have led to an overestimate of subjects’ exposures to motor vehicle and road dust sources and an underestimate of health effects; that is, it is possible that levels lower than those used have significant adverse effects on respiratory health of children with asthma in our region. Determination of spatial variability of PM_2.5_ away from central site monitors will be needed to improve assessment of exposures actually experienced by vulnerable populations.

## Conclusions

The composition of ambient fine particles is complex, because it depends on emission sources and atmospheric processes. The U.S. EPA national air quality standard for PM_2.5_ considers only mass and not composition (U.S. EPA 2009). However, health impacts associated with exposures to specific particle components or sources, especially for vulnerable populations, may be missed with this aggregate characterization (Franklin et al. 2008; Sarnat et al. 2008). Apportioning fine particle mass according to contributing sources may help unmask source-specific associations. For children with asthma living in an area of noncompliance with PM_2.5_ standards, increased risk of daily symptoms and medication use was associated with daily traffic-related fine particle sources.

## Figures and Tables

**Table 1:** Personal characteristics for children participating in a year-long prospective study (*n =* 149), New Haven, Connecticut, August 2000–January 2004.

Enrollment characteristic	No. (%)
Sex	
Male	89 (59.7)
Female	60 (40.3)
Ethnicity	
White	84 (56.4)
Black	36 (24.2)
Hispanic	27 (18.1)
Other	2 (1.3)
Age at enrollment (years)	
< 8	70 (47.0)
≥ 8	79 (53.0)
Mother’s education (years)^a^	
< 12	13 (8.7)
12–15	83 (55.7)
> 15	53 (36.0)
Environmental exposure to tobacco smoke	
No	140 (94.0)
Yes	9 (6.0)

a The adult respondent was the subject’s mother in all but two homes.

**Table 2:** Distribution of daily symptoms, daily inhaler use, and mean asthma severity during a year-long prospective study (*n =* 149), New Haven, Connecticut, August 2000–January 2004.

Variable	No. (%)
Symptoms and inhaler use during 12-month study period (days)	
Wheeze	
None	35 (23.5)
1–7	36 (24.2)
8–14	22 (14.8)
15–21	15 (10.1)
22–29	11 (7.4)
≥ 30	30 (20.1)
Persistent cough	
None	20 (13.5)
1–7	31 (21.0)
8–14	12 (8.1)
15–21	17 (11.5)
22–29	21 (14.2)
≥ 30	47 (31.8)
Shortness of breath	
None	46 (30.9)
1–7	43 (28.9)
8–14	23 (15.4)
15–21	9 (6.0)
22–29	9 (6.0)
≥ 30	19 (12.8)
Chest tightness	
None	49 (32.9)
1–7	44 (29.5)
8–14	20 (13.4)
15–21	9 (6.0)
22–29	7 (4.7)
≥ 30	20 (13.4)
Short-acting inhaler use	
None	29 (19.5)
1–7	28 (18.8)
8–14	14 (9.4)
15–21	11 (7.4)
22–29	11 (7.4)
≥ 30	56 (37.6)
Asthma severity (GINA score)^a^	
0 No symptoms/medication	5 (3.4)
1 Intermittent	66 (44.9)
2 Mild persistent	29 (19.7)
3 Moderate persistent	30 (20.4)
4 Severe persistent	17 (11.6)

a A five-point asthma severity score [from 0 (no symptoms or medication use) to 4 (severe persistent)], based on the Global Initiative for Asthma guidelines (U.S. Department of Health and Human Services 2002) was calculated for each month of the study. The mean severity was the arithmetic mean of the 12 monthly severity scores.

**Table 3:** Source contributions^a^ to PM_2.5_ mass (μg/m^3^) and elemental (ng/m^3^) concentrations in New Haven, Connecticut, August 2000 – January 2004.

	Source						Mass		
Source	Motor vehicles	Road dust	Sulfur	Biomass burning	Oil	Sea salt	Estimated	Measured	%RMSE
Element (ng/m^3^)									
EC	961.3^b^	403.4	118.8	22.1	68.1	–19.0	1554.8	1894.5	17.9
Zn	22.3^b^	2.6	0.8	0.5	1.7	1.0	29.0	23.0	26.3
Pb	3.4^b^	0.5	0.6	0.5	—	0.1	5.1	4.9	4.0
Cu	2.1^b^	1.3	0.5	1.8	0.2	—	6.0	6.3	5.5
Se	0.4^b^	–0.1	0.2	—	0.0	0.0	0.5	0.5	3.5
Si	–4.5	115.7^b^	5.7	4.8	2.0	2.2	125.9	117.0	7.6
Fe	84.7	101.1^b^	3.0	11.6	12.2	–1.7	210.9	219.0	3.7
Al	–7.0	54.8^b^	7.0	5.8	—	1.6	62.0	67.5	8.1
Ca	17.8	29.1^b^	—	1.5	1.1	2.0	51.6	51.2	0.7
Ba	2.9	4.9^b^	0.5	4.1	0.8	–0.4	12.7	12.6	0.4
Ti	1.2	4.2^b^	0.5	0.9	—	—	6.9	7.4	6.3
S	210.4	166.3	906.7^b^	75.4	18.7	26.0	1403.4	1451.8	3.3
P	14.0	6.6	36.8^b^	2.6	1.5	—	61.5	64.8	5.2
K	–8.0	13.8	12.4	60.5^b^	–2.7	3.7	79.6	63.2	25.8
V	0.6	1.1	0.8	0.2	6.0^b^	0.2	8.9	9.9	9.4
Ni	1.9	0.4	0.2	0.1	2.6^b^	0.2	5.6	5.2	6.9
Na	20.9	29.3	37.0	4.4	—	47.0^b^	140.1	180.8	22.5
Cl	20.5	—	–11.2	—	6.9	32.0^b^	48.7	29.6	64.4
PM_2.5_ (μg/m^3^)	6.6^b^	2.3	5.5	0.9	0.8	0.5	16.6	17.0	2.4
Daily source contribution to PM_2.5_ (%)									
Median	42.5	12.3	28.7	4.4	2.2	1.2			
IQR	26.7	13.8	30.3	5.6	7.7	7.2			

— nonsignificant contribution.

a Mean concentration for 1,181 days between August 2000 and January 2004.

b Maximum contribution from a particular source for each element.

**Table 4:** ORs from separate repeated measures logistic regression analyses^a^ of associations between daily respiratory symptoms and each daily elemental concentration of PM_2.5_.

			Wheeze		Persistent cough		Shortness of breath		Chest tightness		Inhaler short-acting	
Source/element	Lag	Unit increase (ng/m^3^ )	OR	*p*-Value	OR	*p*-Value	OR	*p*-Value	OR	*p*-Value	OR	*p*-Value
Motor vehicles												

EC	L0	1,000	1.04	0.04	1.01	0.42	1.06	0.001	1.03	0.20	1.01	0.15
	L02	1,000	1.07	0.06	1.03	0.23	1.12	0.01	1.10	0.04	1.02	0.40
Zn	L0	10	1.00	0.69	1.00	0.60	1.02	0.001	1.00	0.72	1.00	0.41
	L02	10	1.00	0.98	1.00	0.94	1.04	0.06	1.03	0.13	1.01	0.53
Pb	L0	5	1.02	0.31	1.02	0.25	1.03	0.11	1.02	0.31	1.01	0.06
	L02	5	1.07	0.13	1.05	0.12	1.12	0.01	1.10	0.02	1.04	0.10
Cu	L0	5	1.01	0.59	1.02	0.13	1.06	0.01	1.03	0.23	1.01	0.22
	L02	5	1.02	0.67	1.05	0.04	1.06	0.21	1.04	0.39	1.01	0.46
Se	L0	1	1.00	0.97	1.00	0.84	1.02	0.40	1.00	0.79	0.99	0.20
	L02	1	1.02	0.71	0.98	0.43	1.02	0.67	0.98	0.61	0.99	0.75

Road dust												

Si	L0	100	1.03	0.03	1.02	0.01	1.04	0.01	1.02	0.20	1.02	0.004
	L02	100	1.07	0.04	1.05	0.02	1.08	0.02	1.06	0.10	1.03	0.09
Fe	L0	100	1.04	0.02	1.02	0.06	1.06	0.002	1.01	0.47	1.02	0.004
	L02	100	1.07	0.05	1.04	0.04	1.08	0.04	1.05	0.21	1.03	0.08
Al	L0	50	1.02	0.17	1.03	0.002	1.05	0.002	1.02	0.21	1.02	0.02
	L02	50	1.07	0.03	1.06	0.01	1.09	0.004	1.07	0.04	1.02	0.11
Ca	L0	50	1.07	0.02	1.05	0.01	1.10	0.002	1.04	0.26	1.04	0.01
	L02	50	1.14	0.04	1.09	0.03	1.18	0.01	1.14	0.07	1.04	0.17
Ba	L0	10	0.99	0.57	1.00	0.83	1.04	0.02	1.01	0.63	1.01	0.08
	L02	10	0.99	0.81	1.00	0.81	1.03	0.38	1.02	0.51	1.01	0.36
Ti	L0	5	1.00	0.59	1.00	0.57	1.01	0.01	1.00	0.34	1.00	0.72
	L02	5	1.01	0.56	1.01	0.29	1.03	0.05	1.01	0.52	1.00	0.66

Sulfur												

S	L0	1,000	0.98	0.43	1.00	0.84	1.01	0.63	0.99	0.80	0.99	0.13
	L02	1,000	1.00	0.99	1.02	0.27	1.01	0.79	1.02	0.68	1.00	0.81
P	L0	50	0.98	0.39	1.00	0.75	1.01	0.61	1.00	0.88	0.98	0.15
	L02	50	0.99	0.89	1.03	0.30	1.01	0.78	1.02	0.67	1.00	0.99

Biomass burning												

K	L0	50	0.98	0.06	1.00	0.64	1.01	0.01	1.01	0.02	1.00	0.68
	L02	50	0.96	0.04	1.00	0.86	1.00	0.79	0.99	0.67	0.99	0.28

Oil												

V	L0	10	0.99	0.73	1.01	0.56	1.01	0.46	0.99	0.71	0.98	0.12
	L02	10	0.93	0.04	0.96	0.05	0.98	0.58	0.94	0.12	0.96	0.03
Ni	L0	5	1.01	0.59	1.01	0.21	1.04	0.05	1.01	0.58	1.01	0.48
	L02	5	0.99	0.72	1.00	0.99	1.04	0.32	1.01	0.84	1.01	0.48

Sea salt												

Na	L0	100	0.98	0.23	1.00	0.58	1.00	0.94	0.99	0.43	0.99	0.35
	L02	100	0.97	0.29	0.98	0.21	0.99	0.74	0.98	0.61	0.99	0.37
Cl	L0	10	1.00	0.89	1.00	0.31	1.00	0.89	1.00	0.24	1.00	0.69
	L02	10	1.00	0.81	1.00	0.06	1.00	0.80	1.00	0.65	1.00	0.83

Associations are shown for same-day exposure (L0) and averaged over the same day and previous 2 days (L02). n = 149 children with asthma, New Haven, Connecticut, August 2000–January 2004.

a Separate analyses were performed for each element and each respiratory symptom and medication use. Models were adjusted for season, day of week, and date. ORs are given for the unit increase given in the second column for each element (and EC). Individual elements in fine particle mass may come from more than one source but are listed here according to their major source. See Table 3 for the contribution of each source to each element’s total mass. Results from analyses using elemental exposures lagged by 1 or 2 days (L1, L2) are included in Supplemental Material, Table 2 (available online at http://www.ehponline.org/members/2009/0800335/suppl.pdf).

**Table 5:** ORs (95% CIs) from repeated measures logistic regression models of respiratory symptoms and rescue medication use and daily source concentrations of PM_2.5_.

Source	Wheeze	Persistent cough	Shortness of breath	Chest tightness	Inhaler short-acting
L0 model					
Motor vehicles	1.05 (0.99–1.10)	1.02 (0.99–1.04)	1.06 (1.01–1.11)	1.02 (0.97–1.08)	1.02 (1.00–1.05)
Road dust	1.10 (1.01–1.19)	1.06 (1.01–1.11)	1.12 (1.02–1.22)	1.04 (0.95–1.15)	1.06 (1.02–1.11)
Sulfur	0.97 (0.94–1.00)	1.00 (0.98–1.01)	0.98 (0.94–1.02)	0.99 (0.94–1.03)	0.98 (0.97–1.00)
Biomass burning	0.80 (0.66–0.98)	0.97 (0.92–1.03)	1.05 (0.95–1.17)	1.06 (0.95–1.18)	1.00 (0.96–1.03)
Oil	1.02 (0.86–1.20)	1.02 (0.95–1.10)	1.07 (0.92–1.26)	0.99 (0.82–1.18)	0.98 (0.91–1.05)
Sea salt	0.96 (0.86–1.07)	0.99 (0.92–1.07)	1.01 (0.92–1.12)	0.95 (0.84–1.08)	0.99 (0.94–1.04)
L02 model					
Motor vehicles	1.10 (1.01–1.19)	1.03 (0.98–1.09)	1.12 (1.01–1.24)	1.08 (0.98–1.20)	1.03 (0.98–1.08)
Road dust	1.26 (1.05–1.51)	1.16 (1.02–1.32)	1.28 (1.05–1.55)	1.20 (0.97–1.49)	1.09 (1.00–1.19)
Sulfur	0.98 (0.92–1.04)	1.01 (0.98–1.05)	0.97 (0.90–1.04)	1.00 (0.92–1.08)	1.00 (0.97–1.03)
Biomass burning	0.64 (0.46–0.88)	0.93 (0.81–1.06)	0.78 (0.52–1.18)	0.87 (0.62–1.22)	0.95 (0.87–1.04)
Oil	0.80 (0.56–1.08)	0.84 (0.71–1.00)	0.94 (0.69–1.29)	0.80 (0.58–1.10)	0.92 (0.81–1.05)
Sea salt	0.91 (0.82–1.16)	0.88 (0.77–1.01)	1.01 (0.79–1.29)	0.95 (0.71–1.27)	0.97 (0.88–1.07)

n = 149 children with asthma, New Haven, Connecticut, August 2000–January 2004. Separate logistic regression analyses were performed for each health outcome. Each model included the six PM_2.5_ sources listed, either as same-day concentrations (L0), or the concentration averaged over the same day and previous 2 days (L02), as well as variables controlling for season, day of week, and date. Logistic regressions were performed using GEE. All ORs are given for a 5-μg/m^3^ increase in source concentration.

**Table 6:** Effect of adding a gaseous co-pollutant to the source exposure model [OR (95% CI)].

		Co-pollutants			
Outcome/source	Sources alone	NO_2_	CO	SO_2_	O_3_
Wheeze					
Motor vehicles	1.05 (0.99–1.10)	1.03 (0.98–1.08)	1.05 (0.99–1.11)	1.04 (0.99–1.09)	1.06 (0.97–1.16)
Road dust	1.10 (1.01–1.19)	1.11 (1.02–1.20)	1.10 (1.01–1.19)	1.10 (1.01–1.19)	1.11 (1.01–1.23)
Sulfur	0.97 (0.94–1.00)	0.96 (0.92–0.99)	0.97 (0.94–1.01)	0.97 (0.93–1.00)	0.95 (0.91–1.00)
Biomass burning	0.80 (0.66–0.98)	0.79 (0.65–0.98)	0.80 (0.66–0.98)	0.79 (0.64–0.98)	0.74 (0.57–0.97)
Oil	1.02 (0.86–1.20)	1.02 (0.87–1.21)	1.02 (0.86–1.20)	1.01 (0.86–1.19)	0.92 (0.62–1.39)
Sea salt	0.96 (0.86–1.07)	0.96 (0.85–1.07)	0.96 (0.86–1.08)	0.95 (0.85–1.07)	1.01 (0.72–1.40)
Co-pollutant		1.08 (0.99–1.18)	1.00 (0.94–1.07)	1.02 (0.96–1.09)	1.08 (0.99–1.18)
Short-acting inhaler use					
Motor vehicles	1.02 (1.00–1.05)	1.02 (0.99–1.04)	1.02 (0.99–1.05)	1.02 (0.99–1.04)	1.02 (0.98–1.07)
Road dust	1.06 (1.02–1.11)	1.06 (1.02–1.10)	1.06 (1.02–1.11)	1.06 (1.02–1.11)	1.06 (1.00–1.13)
Sulfur	0.98 (0.97–1.00)	0.98 (0.96–1.00)	0.98 (0.96–1.00)	0.98 (0.96–1.00)	0.97 (0.95–1.00)
Biomass burning	1.00 (0.96–1.03)	1.00 (0.96–1.03)	0.99 (0.96–1.03)	0.99 (0.96–1.03)	0.99 (0.95–1.03)
Oil	0.98 (0.91–1.05)	0.98 (0.91–1.05)	0.97 (0.91–1.04)	0.97 (0.91–1.04)	1.03 (0.88–1.22)
Sea salt	0.99 (0.94–1.04)	0.99 (0.94–1.04)	0.99 (0.94–1.04)	0.99 (0.94–1.04)	1.01 (0.88–1.15)
Co-pollutant		1.01 (0.97–1.06)	1.02 (0.98–1.05)	1.01 (0.99–1.04)	1.01 (0.97–1.05)

*n* = 149 children with asthma, New Haven, Connecticut, August 2000–January 2004. Separate repeated measures logistic regression analyses using GEE were performed for each health outcome. Each model includes the six particle sources (same-day exposure) as well as season, day of week, and date. Gaseous co-pollutants were added to the source exposure model one at a time. ORs for sources are given for a 5-μg/m^3^ increase in source concentration; ORs for co-pollutants are given for each 20 ppb (NO_2_, ozone), 0.5 ppm (CO), or 5 ppb (SO_2_). Pearson correlations between PM_2.5_ sources and criteria pollutants were < 0.20 except for motor vehicles with NO_2_ (*r* = 0.49), CO (*r* = 0.59), SO_2_ (*r* = 0.45); road dust with NO_2_ (*r* = 0.37), O_3_ (*r* = 0.58); and oil with CO (*r* = 0.22), SO_2_ (*r* = 0.32).
